# Tumor-targeting *Salmonella typhimurium* A1-R in combination with doxorubicin eradicate soft tissue sarcoma in a patient-derived orthotopic xenograft (PDOX) model

**DOI:** 10.18632/oncotarget.7226

**Published:** 2016-02-07

**Authors:** Takashi Murakami, Jonathan DeLong, Fritz C. Eilber, Ming Zhao, Yong Zhang, Nan Zhang, Arun Singh, Tara Russell, Samantha Deng, Jose Reynoso, Cuong Quan, Yukihiko Hiroshima, Ryusei Matsuyama, Takashi Chishima, Kuniya Tanaka, Michael Bouvet, Sant Chawla, Itaru Endo, Robert M. Hoffman

**Affiliations:** ^1^ AntiCancer, Inc., San Diego, California, USA; ^2^ Department of Surgery, University of California, San Diego, California, USA; ^3^ Department of Gastroenterological Surgery, Graduate School of Medicine, Yokohama City University, Yokohama, Japan; ^4^ Division of Hematology-Oncology, University of California, Los Angeles, California, USA; ^5^ Division of Surgical Oncology, University of California, Los Angeles, California, USA; ^6^ Sarcoma Oncology Center, Santa Monica, California, USA

**Keywords:** nude mice, patient-derived orthotopic xenograft, soft tissue sarcoma, Salmonella typhimurium A1-R, tumor-targeting

## Abstract

A patient with high grade undifferentiated pleomorphic soft-tissue sarcoma from a striated muscle was grown orthotopically in the right biceps femoris muscle of mice to establish a patient-derived orthotopic xenograft (PDOX) model. Twenty PDOX mice were divided into 4 groups: G1, control without treatment; G2, *Salmonella typhimurium* (*S. typhimurium*)A1-R administered by intratumoral (i.t.) injection once a week for 4 weeks; G3, doxorubicin (DOX) administered by intraperitoneal (i.p.) injection once a week for 4 weeks; G4, *S. typhimurium* A1-R (i.t.) administered once a week for 2 weeks followed by i.p. doxorubicin once a week for 2 weeks. On day 25 from the initiation of treatment, tumor volume in G2, G3, and G4 was significantly lower than G1. Mice found without gross tumor included one mouse (20%) in G2; one mouse (20%) in G3; and 3 mice (60%) in G4. Body weight loss did not significantly differ between the 3 treated groups or from the untreated control. Histological examination revealed eradication of tumor only in G4 where mice were treated with *S. typhimurium* A1-R followed by DOX. Our present study indicates future clinical potential of combining *S. typhimurium* A1-R with chemotherapy such as DOX for soft tissue sarcoma patients.

## INTRODUCTION

For soft-tissue sarcoma, the 5-year survival for patients with metastatic disease is less than 20% [1, 2]. Doxorubicin (DOX) treatment for advanced soft tissue sarcoma resulted in a median survival of only 18 months. Therefore, development of improved treatment is needed.

Records for almost 300 years have documented cancer patients going into remission after a bacterial infection if they survived the infection [3]. For example, in 1867, Busch reported that a malignant tumor disappeared when the patient contracted what is now known as *Streptococcus pyogenes* [4].

In the late nineteenth century, William B. Coley at Memorial Hospital in New York, the precursor of Memorial Sloan-Kettering Cancer Center, treated cancer patients with *S. pyogenes*. Coley's first patient infected with *S. pyogenes* recovered from head and neck cancer. Coley injected many cancer patients with *S. pyogenes* and often had good results [3].

*Salmonella typhimurium (S. typhimurium)*, is a facultative anaerobe which confers important advantages as a potential cancer therapeutic, in that a facultative anaerobe can grow in the oxic viable region of tumors as well as necrotic regions [5]. In a Phase I clinical trial on patients with metastatic melanoma and renal carcinoma, the *S. typhimurium* strain tested (VNP20009), attenuated by msbB and purI mutations, was safely administered to patients [6].

The tumor-targeting *S. typhimurium* A1-R strain developed by our laboratory has higher tumor-colonization efficacy and antitumor efficacy than *S. typhimurium*-VNP20009 [7], possibly because it has fewer attenuation mutations. *S. typhimurium* A1-R is auxotrophic for Leu—Arg, which prevents it from mounting a continuous infection in normal tissues. *S. typhimurium* A1-R was able to inhibit or eradicate primary and metastatic tumors as monotherapy in nude mouse models of prostate [8, 9], breast [10-12], lung [13, 14], pancreatic [15-19], ovarian [20, 21] stomach [22], and cervical cancer [23], as well as sarcoma cell lines [24-26] and glioma [27, 28], all of which are highly aggressive tumor models.

Previously, we developed a patient-derived orthotopic xenograft (PDOX) nude-mouse model of soft-tissue sarcoma. The sarcoma was resistant to gemcitabine. Pazopanib tended to reduce the tumor volume compared to the untreated mice, but there was no significant difference. However, *S. typhimurium* A1-R significantly inhibited tumor growth compared to the untreated mice. These results suggest tumor-targeting *S. typhimurium* A1-R is a promising treatment for chemo-resistant soft-tissue sarcoma [29].

A human patient with advanced leiomyosarcoma was treated with an intratumoral injection of *Clostridium novyi* (*C. novyi*-NT) spores which reduced the tumor within and surrounding the bone [30].

Since *S. typhimurium* A1-R is a facultative anaerobe, unlike *C. novyi*-NT, which is an obligate anaerobe, it was thought to have more broad application for cancer therapy. In addition, since *S. typhimurium* A1-R can greatly potentiate cytotoxic chemotherapy and the fact that DOX is first-line therapy for soft tissue sarcoma, it was appropriate to determine the efficacy of *S. typhimurium* A1-R followed by DOX in a PDOX model of soft tissue sarcoma compared to either therapy alone.

## RESULTS AND DISCUSSION

### Effects of single agent and combined *S. typhimurium* A1-R and DOX on sarcoma PDOX growth

On the 25^th^ day from initiation of treatment, tumor volume was significantly smaller in the animals treated with *S. typhimurium* A1-R (G2) (245.3 ± 141.6 mm^3^, *p* < 0.01); the animals treated with DOX (G3) (165.5 ± 247.7 mm^3^, *p* < 0.05); and the animals treated with *S. typhimurium* A1-R followed by DOX (G4) (138.4 ± 209.3 mm^3^, *p* < 0.01) compared to the untreated control animals (G1) (1460.3 ± 1136.6 mm^3^, Figure 2). There were no significant differences in tumor volume between G2, G3, and G4. One tumor in G2, 1 tumor in G3, and 3 tumors in G4 achieved complete remission (no detectable tumor). Mouse body weight did not significantly differ between the 3 treatment groups, and the treatment groups did not significantly differ in body weight from the untreated control group. Since the tumor diameter in G1 reached 20 mm after the 25^th^ treatment day, all mice in G1 were sacrificed by the 29^th^ day.

**Figure 1:**
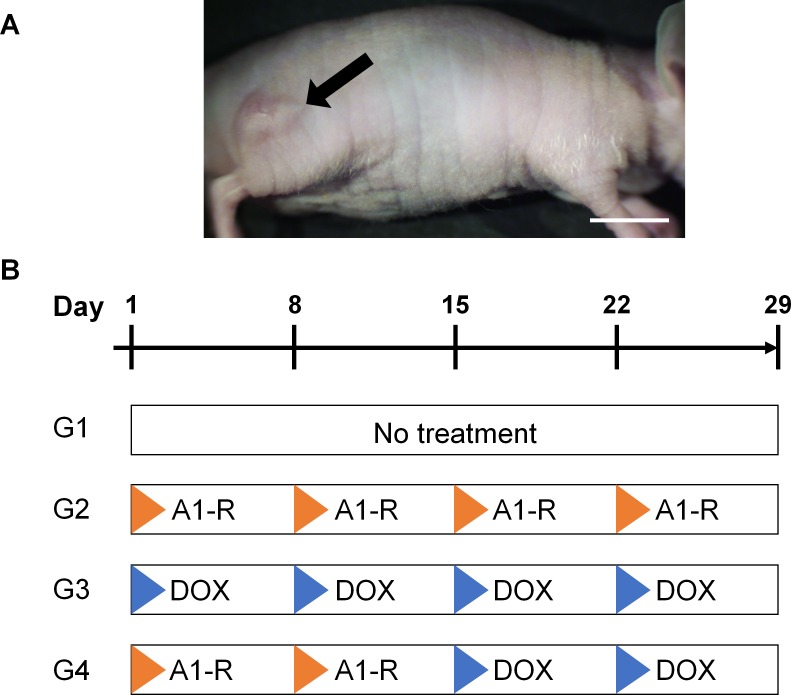
Treatment model and protocol **A.** Soft tissue sarcoma PDOX mouse model. Ten days after surgical orthotopic implantation (SOI), tumors in the right biceps femoris muscle had grown more than 5 mm in diameter. Arrow indicates a grown tumor. Scale bar: 10 mm. **B.** Treatment protocol. G1: untreated control; G2: treated with *S. typhimurium* A1-R (A1-R)-alone (i.t., 5 × 10^7^ CFU/body, weekly, 4 weeks) G3: treated with doxorubicin (DOX)-alone (i.p., 3 mg/kg, weekly, 4 weeks); G4: treated with *S. typhimurium* A1-R (i.t., 5 × 10^7^ CFU/body, weekly, 2 weeks) followed by DOX (i.p., 3 mg/kg, weekly, 2 weeks). Each group contained 5 mice.

**Figure 2:**
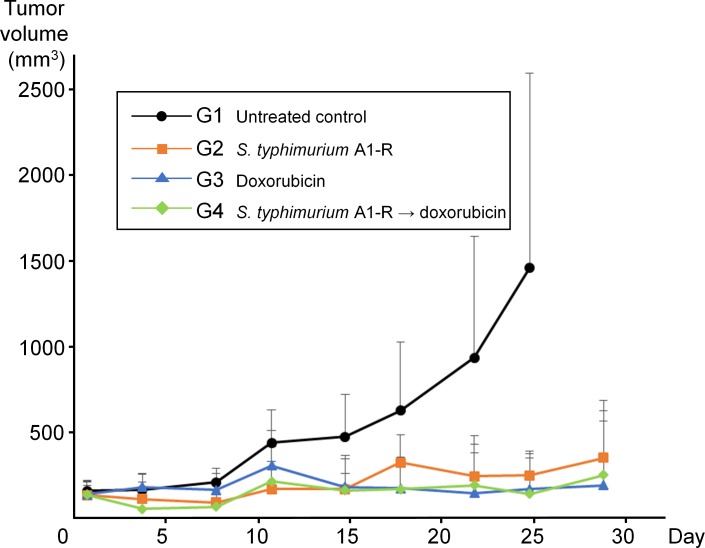
*S. typhimurium* and A1-R doxorubicin or their combination significantly inhibited tumor growth in a soft tissue sarcoma PDOX model Graph shows tumor volume at each time point. On day 25 from initial treatment, tumor volume in G2 (*S. typhimurium* A1-R-alone) (245.3 ± 141.6 mm^3^, *p* < 0.01); G3 (DOX-alone) (165.5 ± 247.7 mm^3^, *p* < 0.05); and G4 (*S. typhimurium* A1-R followed by DOX) (138.4 ± 209.3 mm^3^, *p* < 0.01) was significantly smaller than in G1. There was not a significant difference between the treated groups. Error bars: + 1 SD.

### Histological response to chemotherapy

**Figure 3:**
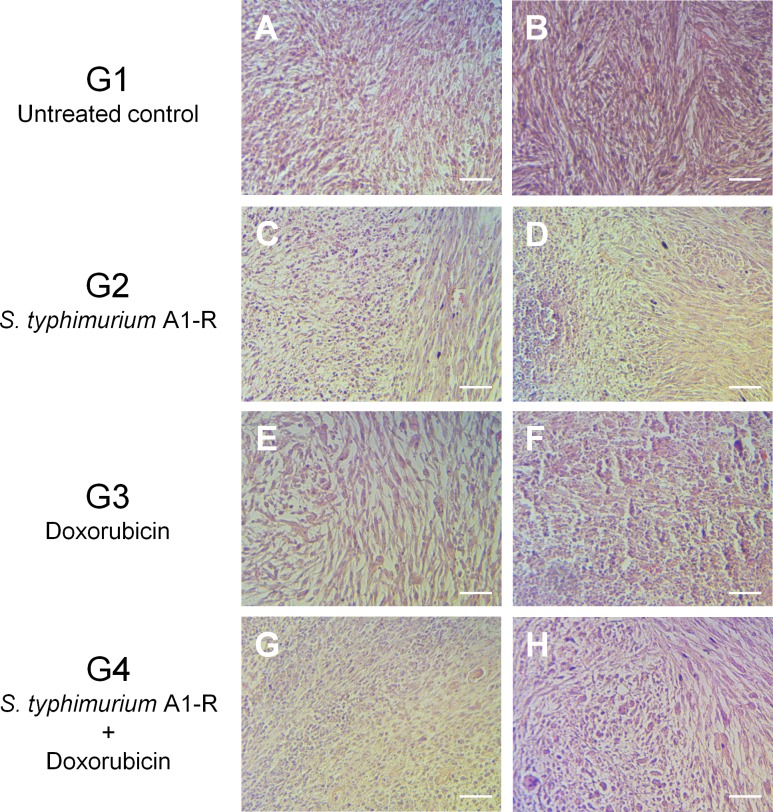
Histological response Untreated tumors (G1) were comprised of spindle-shaped viable sarcomatous cells without necrosis or inflammatory change **A. B.** Tumors treated with *S. typhimurium* A1-R (G2) had components of inflammation (**C.** Grade IIB), or necrosis (**D.** Grade IIA). One of the tumors treated with doxorubicin (DOX) (G3) showed no necrosis (**E.** Grade I), while another tumor showed complete tumor necrosis (**F.** Grade IV). Tumors treated with the combination of *S. typhimurium* A1-R and DOX were destroyed and replaced with inflammatory-type cells (**G. H.** Grade III). Hematoxylin and eosin staining. Scale bars: 100 μm.

Tumors were mainly comprised of viable spindle shaped cells in the tumors from the untreated animals (G1) (Figure 3A, 3B). In G2 treated with *S. typhimurium* A1-R forty to 60% of the tumor tissue consisted of viable tissue (Grade IIA or IIB), whereas the other part was replaced by necrosis or inflammatory cells (Figure 3C, 3D). The response of tumors treated with DOX alone (G3) varied widely; one tumor showed no necrosis (Grade I; Figure 3E), two tumors had a small amount of necrosis (Grade IIA), and the other tumor was necrotic without viable cells (Grade IV; Figure 3F). The two tumors which did not undergo complete remission in G4 were comprised of large areas of necrosis or inflammation (Grade III; Figure 3G, 3H). Tumors in G4, treated with the combination of *S. typhimurium* A1-R followed by DOX were considered eradicated. These results were summarized in Table 1.

**Table 1:** Treatment response between groups

	Histological response (Evans grading system)					
	Grade I	Grade IIA	Grade IIB	Grade III	Grade IV	CR
G1 (n=5)	5	0	0	0	0	0
G2 (n=5)	0	2	2	0	0	1
G3 (n=5)	1	2	0	0	1	1
G4 (n=5)	0	0	0	2	0	3

G1: Untreated control

G2: *S. typhimurium* A1-R-alone

G3: Doxorubicin-alone

G4: *S. typhimurium* A1-R followed by doxorubicin

Recently we established a soft tissue sarcoma PDOX model in which the mouse-grown tumors more closely recapitulated the human histology, as compared to xenografts [31] implanted subcutaneously. This may be a general phenomenon for all tumor types [32].

The higher rate eradication of tumors by the combination of *S. typhimurium* and DOX may be attributable to the “decoy” effect of *S. typhimurium* A1-R [33], which induces quiescent cells to cycle, there by making them more susceptible to chemotheraphy than when the cancer cells are in G_1_/G_0_ which are the majority of cells in a solid tumor [34]. Future experiments will investigate this possibility.

Recently, Roberts et al. [30] treated a patient with advanced leiomyosarcoma with spores of the obligate anaerobe *C. novyi*-NT. A single metastasis was injected with the *C. novyi-*NT spore and appeared to be completely destroyed. The patient died due to other lesions of the leiomyosarcoma. The fact that *C. novyi-*NT is an obligate anaerobe may preclude its systemic administration and may limit its treatment to i.t. injection. This should not be the case for the facultative anaerobe *S. typhimurium* A1-R, where we have shown in previous experiments to be effective against tumors when administered i.v. Although, i.t. administration of *S. typhimurium* A1-R was used in the present experiment as a model for clinical testing, future treatment of soft tissue sarcoma will utilize i.v. administration as well. Future experiments will test simultaneous treatment of *S. typhimurium* A1-R and DOX to determine synergy.

## CONCLUSIONS

The combination of *S. typhimurium* A1-R and subsequent DOX eradicated a soft tissue sarcoma in a PDOX mouse model. In the early part of the 20^th^ century, bacterial therapy of sarcoma was first-line therapy under William B. Coley at the Memorial Hospital of New York. Since DOX is presently considered first-line therapy of soft tissue sarcoma, the combination of DOX with *S. typhimurium* A1-R has important future clinical potential, which we hope can be realized in the near future. Another potential future clinical strategy is to combine sarcoma surgery with adjuvant *S. typhimurium* A1-R which we have shown to be highly effective in mouse models [35].

## MATERIALS AND METHODS

### Mice

Athymic *nu/nu* nude mice (AntiCancer Inc., San Diego, CA), 4-6 weeks old, were used in this study. All animal studies were conducted with an AntiCancer Institutional Animal Care and Use Committee (IACUC)-protocol specifically approved for this study and in accordance with the principals and procedures outlined in the National Institute of Health Guide for the Care and Use of Animals under Assurance Number A3873-1. In order to minimize any suffering of the animals, the use of anesthesia and analgesics were used for all surgical experiments. Animals were anesthetized by subcutaneous injection of a 0.02 ml solution of 20 mg/kg ketamine, 15.2 mg/kg xylazine, and 0.48 mg/kg acepromazine maleate. The response of animals during surgery was monitored to ensure adequate depth of anesthesia. The animals were observed on a daily basis and humanely sacrificed by CO_2_ inhalation when they met the following humane endpoint criteria: severe tumor burden (more than 20 mm in diameter), prostration, significant body weight loss, difficulty breathing, rotational motion and body temperature drop. Animals were housed in a barrier facility on a high efficiency particulate arrestance (HEPA)-filtered rack under standard conditions of 12-hour light/dark cycles. The animals were fed an autoclaved laboratory rodent diet.

### Patient-derived tumor

A large right thigh high-grade undifferentiated pleomorphic sarcoma was resected by F.C.E., Division of Surgical Oncology, University of California, Los Angeles (UCLA). The patient did not receive any chemotherapy or radiotherapy prior to surgery. Written informed consent was obtained from the patient as part of a UCLA Institutional Review Board (IRB #10-001857) approved protocol.

### Establishment of a PDOX model of soft tissue sarcoma by surgical orthotopic implantation (SOI)

A fresh sample of a soft tissue sarcoma which had grown in striated muscle of a patient was obtained and transported immediately to the laboratory at AntiCancer, Inc., on wet ice. The sample was cut into 5-mm fragments and implanted subcutaneously in nude mice. After three weeks, the subcutaneously-implanted tumors grew to more than 10 mm in diameter. The subcutaneously-grown tumors were then harvested and cut into small fragments (3 mm^3^). After the mice were anesthetized with the ketamine solution described above, a 5-mm skin incision was made on the right high thigh into the biceps femoris, which was split to make space for the sarcoma tissue fragment. A single tumor fragment was implanted orthotopically into the space to establish the PDOX model. The wound was closed with a 6-0 nylon suture (Ethilon, Ethicon, Inc., NJ, USA).

### Preparation and administration of *S. typhimurium* A1-R

GFP-expressing *S. typhimurium* A1-R bacteria (AntiCancer, Inc., San Diego, CA, USA) were grown overnight on LB medium and then diluted 1:10 in LB medium. Bacteria were harvested at late-log phase, washed with PBS, and then diluted in PBS. *S. typhimurium* A1-R was injected intratumorally. A total of 5 × 10^7^ CFU *S. typhimurium* A1-R in 50 μl PBS was administered to each tumor.

### Treatment study design in the PDOX model of soft tissue sarcoma

Ten days after orthotopic implantation, tumors reached 5 mm in diameter (Figure 1A). Twenty PDOX model mice were randomized into 4 groups of 5 mice each (Figure 1B): G1, control without treatment; G2, treated with *S. typhimurium* A1-R; i.t. once a week for 4 weeks; G3, treated with 3 mg/kg doxorubicin (pfizer, NewYork, NY,) i.p. once a week for 4 weeks, G4: treated with *S. typhimurium* A1-R (i.t.) once a week for 2 weeks followed by doxorubicin i.p. once a week for 2 weeks. Tumor length, width and mouse body weight were measured twice in a week. Tumor volume was calculated with the following formula: Tumor volume (mm^3^) = length (mm) × width (mm) × width (mm) × 1/2. Data are presented as mean ± SD. When a tumor was not detectible, the tumor response was considered as complete remission. All treated mice were sacrificed on day 29, and tumors were resected for further histological evaluation.

### Histological examination

Fresh tumor samples were fixed in 10% formalin and embedded in paraffin before sectioning and staining. Tissue sections (5 μm) were deparaffinized in xylene and rehydrated in an ethanol series. Hematoxylin and eosin (H &E) staining was performed according to standard protocol. Histological examination was performed with a BHS System Microscope (Olympus Corporation, Tokyo, Japan). Images were acquired with INFINITY ANALYZE software (Lumenera Corporation, Ottawa, Canada). Histological response was evaluated according to the Evans grading system that had been developed for human-cancer histological-response evaluation after chemo-radiotherapy [36]. Briefly, histological response was classified as follows; Grade I: no or little (< 10%) cancer-cell destruction; Grade IIA: 10-49% cancer-cell destruction; Grade IIB: 50-89% cancer cell destruction; Grade III: few (< 10%) viable cancer cells are present, Grade IV: no viable tumor is detectible.

### Statistical analysis

SPSS statistics version 21.0 was used for all statistical analyses (IBM, New York City, NY, USA). Significant differences for continuous variables were determined using the Mann-Whitney U test. A probability value of *P* < 0.05 was considered statistically significant.
